# Case report – Acute Corneal Subepithelial Hydrops (ACSH) during Micropulse Transscleral Cyclophotocoagulation (MPTSC)

**DOI:** 10.1186/s12886-020-01669-6

**Published:** 2020-10-14

**Authors:** Poemen P. Chan, Matthew C.W. Lam, Nafees Baig

**Affiliations:** 1 Department of Ophthalmology & Visual Sciences, The Chinese University of Hong Kong, 4/F, Hong Kong Eye Hospital, 147K Argyle Street, Kowloon, Hong Kong SAR People’s Republic of China; 2 Hong Kong Eye Hospital, Kowloon, Hong Kong SAR People’s Republic of China; 3 Department of Ophthalmology, Hong Kong Sanatorium & Hospital, Hong Kong, People’s Republic of China

**Keywords:** ACSH case report, Acute corneal subepithelial hydrops, MPTSC micropulse transscleral cyclophotocoagulation

## Abstract

**Background:**

To present an unusual intra-operative complication of micropulse transscleral cyclophotocoagulation (MPTSC).

**Case presentation:**

A 72-year old Chinese gentleman, who had primary angle closure glaucoma and had received bilateral laser iridotomy, presented with progressive left eye blurred vision (visual acuity of 20/40 OD and 20/200 OS). Examination reviewed left eye central retinal venous occlusion. The intraocular pressure (IOP) was 19 mmHg OS and was on maximally tolerated topical medications. Four weeks later, the left eye was complication by neovascular glaucoma; the IOP was raised to 26 mmHg despite additional oral acetazolamide and remained elevated after pan-retinal photocoagulation as well as cataract extraction by phacoemulsification. MPTSC was performed 8 days after the phacoemulsification. During the procedure, a sudden protrusion was formed on the corneal surface. On-table examination with operating microscope and portable slit-lamp reviewed an intact corneal epithelium with a globular-shaped collection of fluid at the subepithelial layer – acute corneal subepithelial hydrops (ACSH). The anterior chamber was formed and the globe was intact. After approximately 10–15 minutes, the swelling spontaneously ruptured and became a corneal epithelial defect. The defect healed on the tenth day after the event with conservative management. There was no irreversible corneal damage and the patient subsequently underwent a successful second MPTSC of the left eye because of poorly controlled IOP.

**Conclusion:**

ACSH is a possible intra-operative complication of MPTSC. We have proposed the possible mechanisms of ACSH. It is best to exercise caution when using MPTSC shortly after any incisional intraocular surgery.

## Background

Micropulse transscleral cyclophotocoagulation (MPTSC; IRIDEX Laser Systems, Mountain View, CA) was approved by the Food and Drug Administration in 2015 as a treatment for glaucoma. The laser system delivers a series of short pulses of laser energy (“on” cycle) followed by pauses (“off” cycle). This cyclical delivery of laser allows energy to build up with each pulse to a photocoagulative state in the pigmented epithelium of the ciliary body, whilst the “off cycle” allows the surrounding tissue to cool down and remain below the photocoagulative threshold and, hence, preventing collateral tissue damage [1].

Compared with continuous-wave diode laser transscleral cyclophotocoagulation (DLTSC), MPTSC has demonstrated comparable efficacy with fewer side effects for treating refractory glaucoma [1–5]. With better safety profile, it was suggested that MPTSC could also be suitable for eyes with good central vision [6] and those with previous keratoplasty [7]. Reported complications of MPTSC included prolong postoperative iritis, cystoid macular oedema, loss of ≥2 lines of visual acuity, and phthisis bulbi [8, 9]. Theoretically, MPTSC could also damage the cornea since the laser probe is applied near the limbus. The eye is especially vulnerable shortly after intraocular surgery when there are wounds at the peripheral cornea. However, severe corneal complications due to MPTSC have not been reported. To our knowledge, this is the first case report of an unusual and a potentially dangerous complication of MPTSC – acute corneal subepithelial hydrops (ACSH).

## Case presentation

The authors obtained approval of the case report by the Research Ethics Committee (Kowloon Central/Kowloon East) of the Hospital Authority, Hong Kong (Ref: KC/KE-19-0256/ER-3). Written informed consent to publish (including individual details and images) was obtained from the patient.

A 72-year old Chinese gentleman, who had chronic obstructive airway disease (COAD) and type II respiratory failure on oxygen therapy, presented with progressive blurring of vision in his left eye. He had previously undergone bilateral peripheral laser iridotomy because of left eye advanced primary angle closure glaucoma (PACG) and right eye primary angle closure (PAC). He had opted for conservative management and had refused further intervention, such as phacoemulsification and intraocular lens (IOL) insertion, because of the poor health status and known guarded left eye visual prognosis.

At presentation, his best-corrected visual acuity was 20/40 OD and 20/200 OS. Intraocular pressure (IOP) was 13 mmHg OD and 19 mmHg OS (all IOPs were measured with Goldmann applanation tonometer unless specified). His left eye was on three topical medications including topical latanoprost, brimonidine, and brinzolamide – β-blockers were contraindicated because of COAD. The cup-to-disc ratio was 0.3 OD and 0.9 OS. Dark-room indirect gonioscopy with single-mirror Goldmann lens showed Shaffer’s grade 1 angle bilaterally. On slit-lamp examination, there was no sign of inflammation, pigment dispersion or neovascularization in the anterior chamber. A left eye relative afferent pupillary defect (RAPD) was present. Dilated fundal examination (with binocular indirect ophthalmoscopy and 20D lens) of the left eye reviewed tortuous vessels with diffuse dot-blot haemorrhages; there was neither neovascularization nor macular oedema. The diagnosis of left eye central retinal venous occlusion (CRVO) was made.

After 4 weeks, IOP of the left eye rose to 26 mmHg and neovascularization of the iris was noted. Topical atropine twice daily and oral acetazolamide 250 mg twice daily were given. One week later, pan-retinal photocoagulation was performed on the left eye on two separate occasions (with an interval of 1 week in between). Phacoemulsification and IOL implantation were also performed under topical anaesthesia 2 months after the presentation. It was performed with bimanual technique through a 3.0 mm clear-corneal wound at the 12 o’clock region and two paracentesis wounds at 3 and 9 o’clock region. After injecting viscoelastic into the anterior chamber and completing a continuous curvilinear capsulorrhexis, phacoemulsification was performed with the stop-and-chop technique and cortical aspiration was performed with bimanual technique. A Tecnis ZCB00 1-Piece Acrylic IOL was then implanted into the lens capsule. Viscoelastic was then removed by aspiration and corneal wounds were sealed by stromal hydration. Mild bleeding from the iris was noted during the operation. Otherwise, it was an uneventful surgery. Topical prednisolone acetate (PRED FORTE®) 1 drop 6 times daily OS and topical levofloxacin (CRAVIT®) 1 drop QID OS were given and the patient was asked to continue with all the IOP-lowering medications. Post-operative day 1 and day 7 showed a formed anterior chamber with mild anterior chamber cell reaction. The IOL was well-placed and stable in the posterior capsule. There was no wound leak and the cornea was clear without corneal oedema. However, left eye IOP remained at 30 mmHg postoperatively despite three topical IOP-lowering eye drops and oral acetazolamide. After a thorough discussion with the patient on the risks and benefits of various glaucoma treatments, MPTSC was performed 8 days after the phacoemulsification and IOL implantation.

**Fig. 1:**
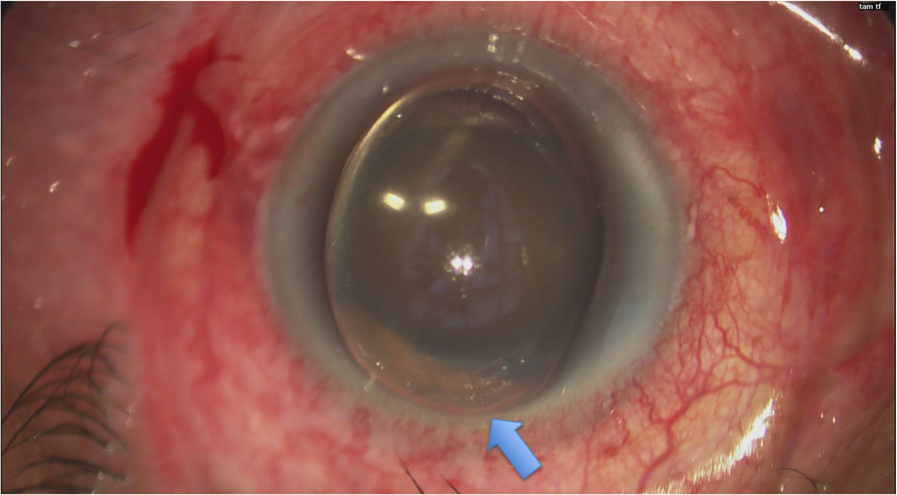
(Surgeon’s view) An acute globular-shaped protrusion from the corneal surface - acute corneal subepithelial hydrops (ACSH). Note the close proximity of the ACSH to the limbus at the superior aspect of the cornea (blue arrow)

The procedure was performed in the operating theatre with retrobulbar anaesthesia (injection of a mixture of 2.5 mL of 0.5% Bupivacaine, 2 mL of 2% Lignocaine and 0.5 mL of 75iu of Hyalase). The laser setting used was 2000 mW of 810 nm infrared diode laser radiation set on micropulse mode (Iris Medical Instruments, Mountain View CA, USA). We planned to deliver the laser for 80 s for each superior and inferior hemisphere, sparing the 3 and 9 o’clock meridians [1]. The probe was applied with firm pressure and was moved in a continuous sliding arc motion along the superior aspect. At the 50th second, when the probe reached the 12 o’clock region, there was a spontaneous formation of a globular-shaped protrusion on the corneal surface (Fig. 1). The procedure was immediately withheld and the eye was examined with an operating microscope.

**Fig. 2:**
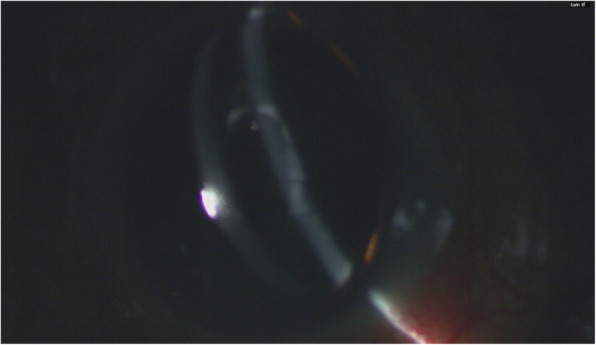
Left eye examined by portable slit-lamp. Patient was in supine position. Oblique slit beam revealed fluid collection at the subepithelial layer. The anterior chamber was formed

**Fig. 3:**
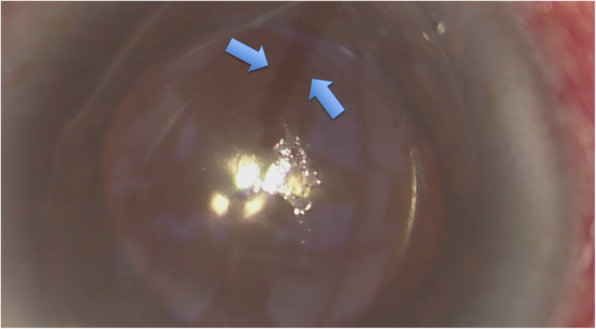
Rupture of the subepithelial hydrops (corneal epithelium was indicated by the blue arrows)

**Fig. 4:**
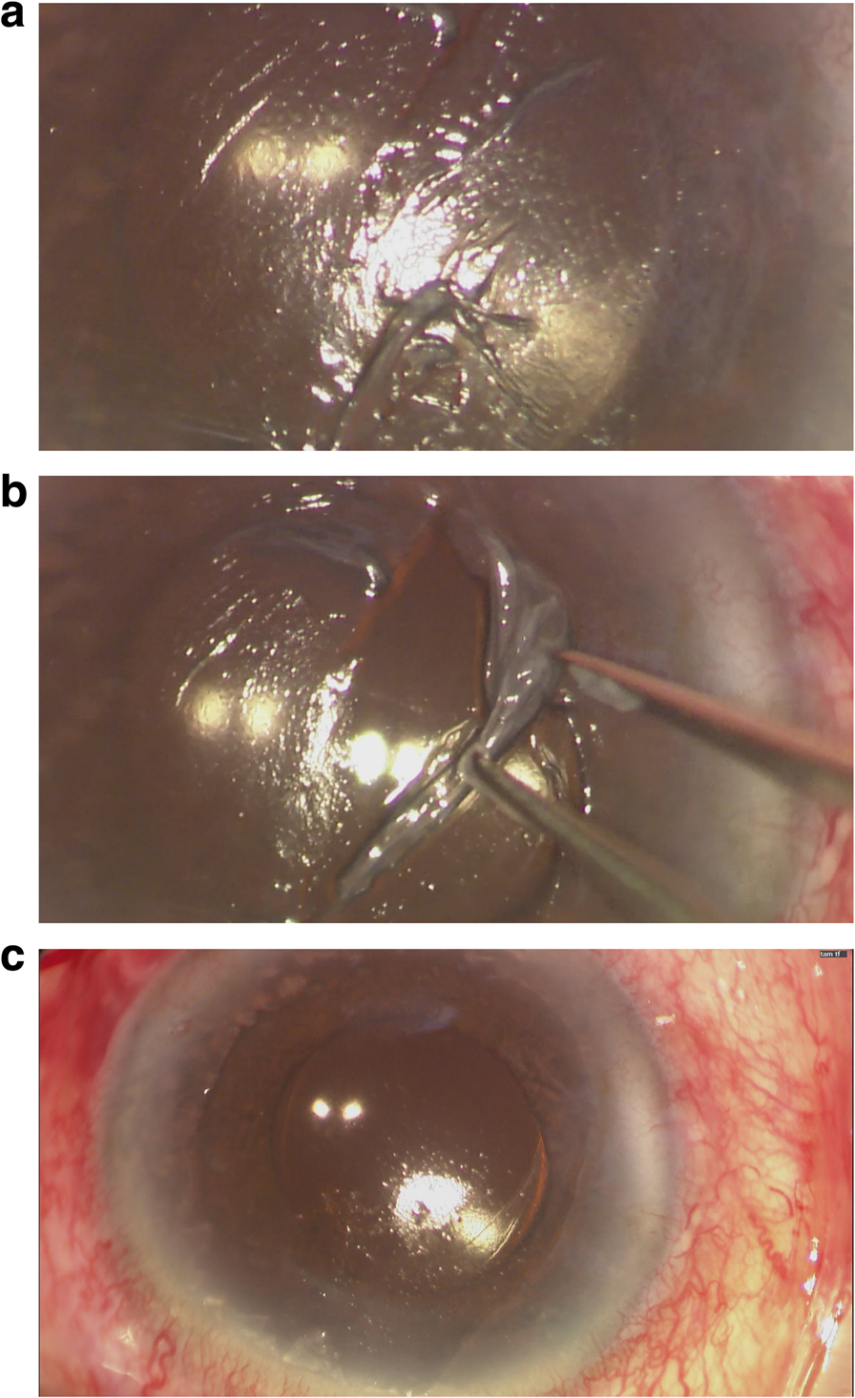
**a** Collapse of the subepithelial hydrops after the rupture, with loose cornea epithelium. **b** Removal of loose epithelium with wet Merocel® sponge and non-toothed forceps. **c**. Status of the cornea after the removal of loose epithelium

As demonstrated in Fig. 1, a globular-shaped hydrops could be identified on the corneal surface. The anterior chamber was formed. Gentle digital palpation on the sclera revealed a firm pressure. Since the patient was lying in a supine posture and it was in the operating theatre setting, the eye was further examined by a portable slit-lamp. Oblique slit beam revealed that the hydrops was at the sub-epithelial level and the epithelium was intact (Fig. 2). The diagnosis of ACSH was made. The corneal subepithelial hydrops then spontaneously ruptured (Fig. 3) in approximately 10–15 min intra-operatively and debridement of the loose epithelium was performed (Fig. 4a-c) – hence, anterior-segment optical coherence tomography (AS-OCT) was not performed to confirm the diagnosis. No leakage was identified (Seidel’s test was negative), the anterior chamber was formed without signs of ruptured globe, and IOP was 23 mmHg (measured with TONO-PEN AVIA®, Reichert Technologies, NY, USA) before the patient left the operating theatre.

The patient was followed up on day one, day three, and day ten after the operation. All IOP-lowering medications (including oral acetazolamide 250 mg BD) were continued. In addition, topical prednisolone acetate (PRED FORTE®) 1 drop BD OS and topical levofloxacin (CRAVIT®) 1 drop QID OS were given. Full corneal epithelialisation was achieved on day ten. The left eye IOP were 15 mmHg, 6 mmHg, and 18 mmHg respectively. The IOP rose to 25 mmHg at 1 month despite maximally tolerated medication. Further MPTSC was subsequently performed in the second month after the last MPTSC and it was uneventful. After this second MPTSC, the IOP was maintained at 15 mmHg with two medications (lantanoprost and brinzolamide), although visual acuity remained the same as before the events (i.e. 20/200 OS).

## Discussion and conclusions

**Fig. 5:**
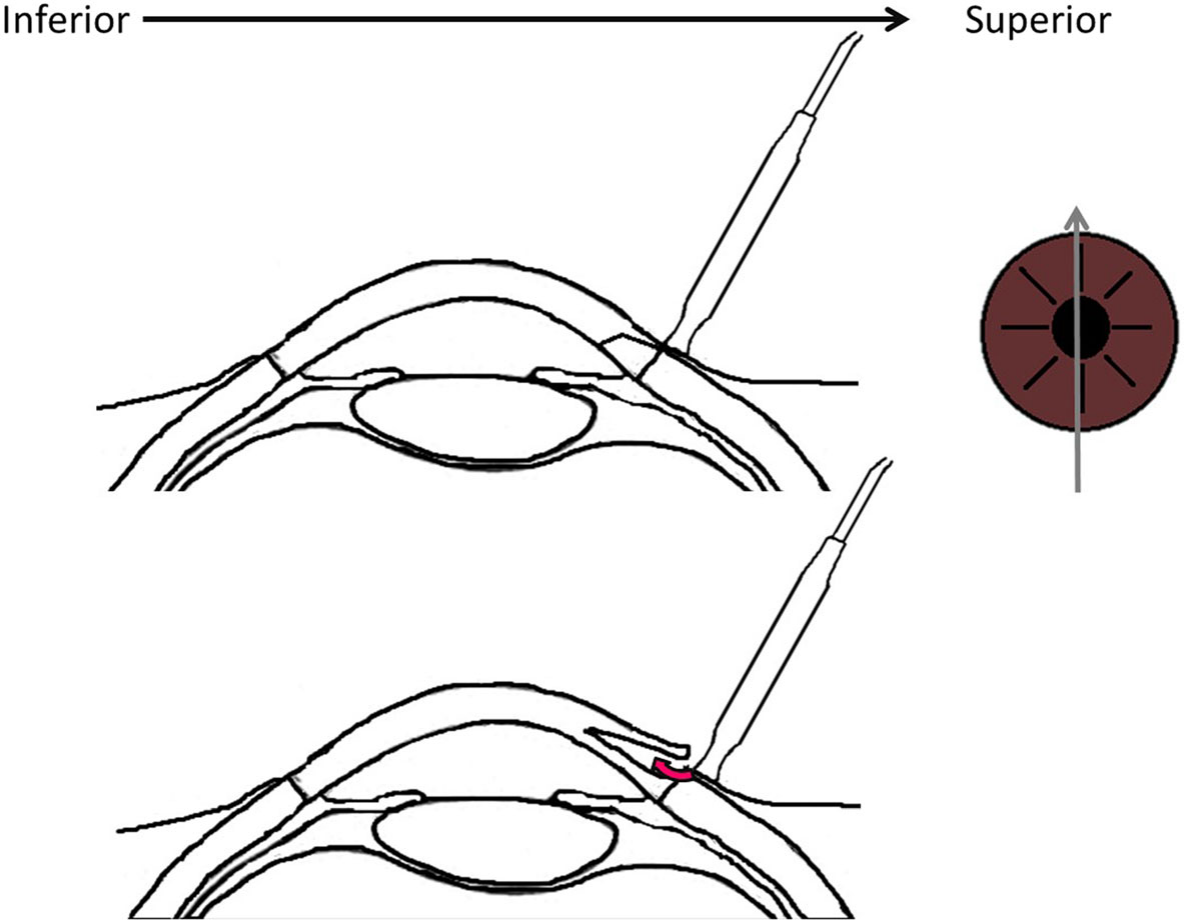
Schematic diagram demonstrating how laser energy could be misdirected towards the corneal wound if the MPTSC probe is not well positioned

To our knowledge, this is the first publication describing an acute corneal complication of MPTSC. A rapid accumulation of fluid at the subepithelial layer led to ACSH. Given that the phacoemulsification was performed through a clear corneal wound at the 12 o’clock region 8 days prior to the MPTSC, we propose that during the MPTSC procedure, the laser probe had undesirably delivered laser energy through the main corneal wound and led to a rapid cleavage along the corneal subepithelial layer. It is possible that, because of the tight palpebral fissure, the MPTSC probe was inadvertently pushed centrally and the laser energy was misdirected towards the corneal wound (Fig. 5 – schematic diagram of the mechanism). Another possible mechanism could be related to the rapid rise in IOP during the procedure. The increased IOP – which could be due to the retrobulbar anaesthesia and/or laser energy of MPTSC – could force the aqueous to areas with recently damaged Descemet’s membrane (i.e. the corneal incision of the cataract surgery). Application of pressure adjacent to the wound might have stretched the wound enough to permit the influx of aqueous into the cornea and resulted in a rapid formation of the ACSH; the temporarily and rapidly elevated IOP increases its imbibition into the corneal epithelial layer through this new channel of disrupted Descemet’s membrane. Either mechanism, by the same token, could have resulted in separation of the cornea at any level and produced different pathologies, such as a rapid and extensive Descemet’s membrane detachment (DMD). Although the patient also has other comorbidities (e.g. COAD, on long-term oxygen, left eye CRVO, left eye neovascular glaucoma), we cannot relate any of these to the occurrence of ACSH.

**Fig. 6:**
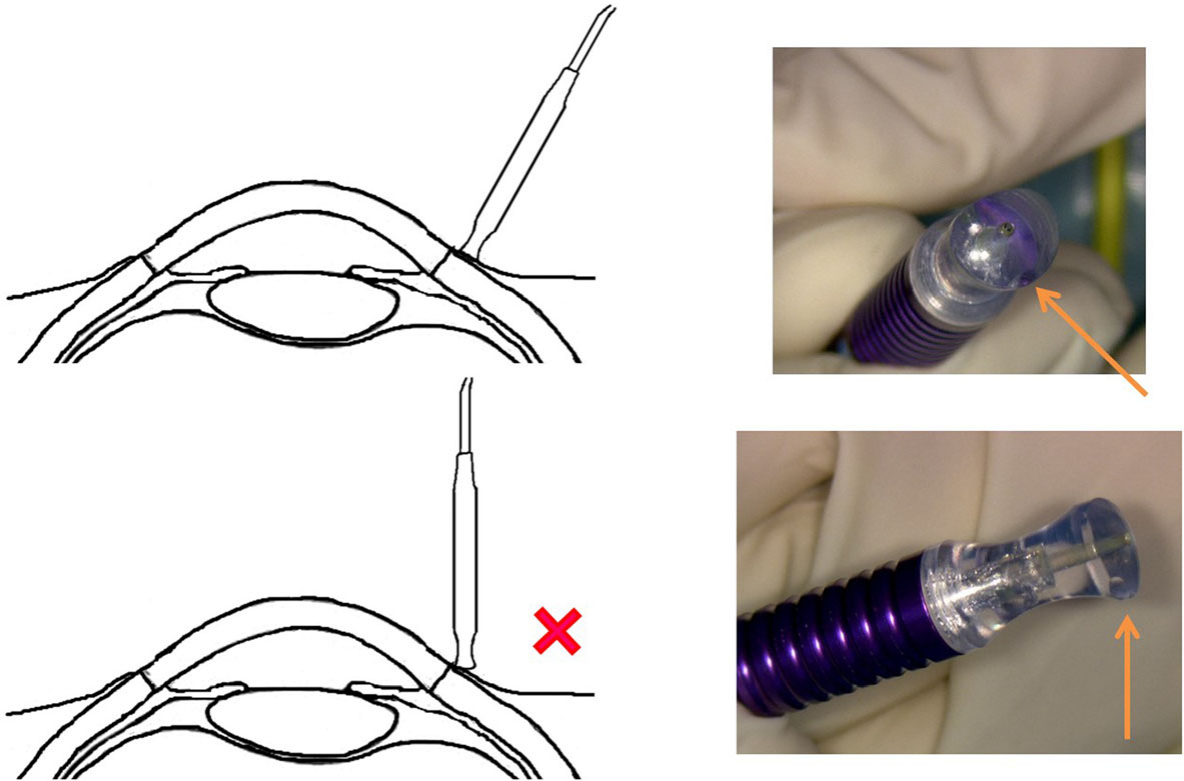
Correct orientation and positioning of MPTSC probe; the notch (orange arrow) should be towards the limbus

**Fig. 7:**
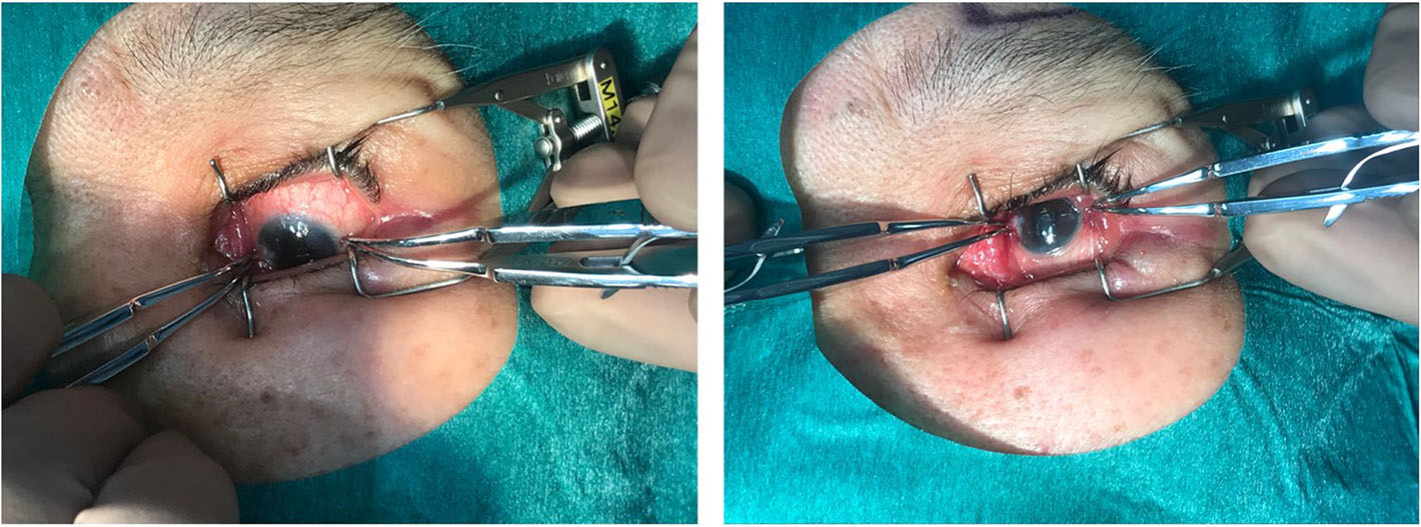
Moody fixation forceps could help mobilize and fix the globe, allowing good exposure for eyes with narrow palpebral fissures

The correct position and orientation of the MPTSC probe during the procedure is important (Fig. 6). Some surgeons prefer performing MPTSC without a lid speculum, because for patients with a tight palpebral fissure, the speculum might obscure the path of the sweeping motion of the probe. Squint hook could be employed to orientate an anesthetised eye, but the globe could slip whilst the probe is moving. Alternatively, two pairs of Moody fixation forceps (held by an assistant) could be used to firmly stabilise the globe for MPTSC (Fig. 7). The forceps can also serve to safeguard the 3 and 9 o’clock region from undesirable laser delivery to the long ciliary nerves and vessels.

Given that the ACSH was likely related to the new clear-corneal wound after the phacoemulsification, we suggest that MPTSC should be performed very carefully soon after any incisional intraocular surgery because of the incomplete healing of the corneal wounds shortly after operation. In a previous study, AS-OCT of the corneal incision demonstrated the presence of localised epithelial bulla, DMD and endothelial gapping 1 day after surgery [10]. Up to 37% of DMD and 20% of endothelial gapping were observed on day eight. In some patients, these could still be detectable 30 days after the operation [11]. During MPTSC, laser energy or the rapidly raised IOP could be distributed to the cornea through the wound gap, especially if the main wound is more peripherally located and if the probe is not well-positioned. In cases where early MPTSC needs to be done shortly after cataract extraction or if MPTSC is combined with cataract surgery, the area of the clear corneal wound should be carefully respected during MPTSC. We repeated the MPTSC uneventfully for the patient two months afterward because we believed that the cornea wound should have been completely healed by then. Pseudophakia is unlikely to be a contraindication for MPTSC, provided that the surgeon is aware of the potential complication. The current study is limited by the fact that this is the only case of ACSH in our centre; we are uncertain whether MPTSC could still lead to ACSH in eyes that had undergone clear-cornea incision months or years beforehand. Similarly, it is also uncertain whether ACSH could develop in corneas that had never undergone any previous surgery. Detailed structure of the ACSH was not evaluated by AS-OCT because the hydrop ruptured soon after it was formed intra-operatively.

We reported a new complication of MPTSC – acute corneal subepithelial hydrop. The case demonstrated that laser energy of MPTSC could be distributed to the cornea and lead to a potentially severe complication. It is important to apply MPTSC with correct positioning and technique, especially shortly after surgery that involved clear cornea wounds.

## Data Availability

Data sharing is not applicable to this article as no datasets were generated or analysed during the current study.

## Abbreviations

ACSH: Acute corneal subepithelial hydrops
AS-OCT: Anterior segment optical coherence tomography
CRVO: Central retinal venous occlusion
DLTSC: Diode laser transscleral cyclophotocoagulation
DMD: Descemet’s membrane detachment
IOL: Intraocular lens
IOP: Intraocular pressure
MPTSC: Micropulse transscleral cyclophotocoagulation
OD: Oculus dexter (i.e. right eye)
OS: Oculus sinister (i.e. left eye)
PAC: Primary angle closure
PACG: Primary angle closure glaucoma
RAPD: Relative afferent pupillary defect
